# Multiple coil closure of isolated aortopulmonary collateral

**DOI:** 10.4103/0974-2069.64357

**Published:** 2010

**Authors:** Sumanta S Padhi, Kinjal D Bakshi, Ram K Shastri

**Affiliations:** Department of Cardiology, Innova Children's Heart Hospital, Secunderabad, India

**Keywords:** Congestive heart failure, isolated aortopulmonary collateral, multiple coil closure, structurally normal heart

## Abstract

A 7-month-old girl was diagnosed to have large aortopulmonary collateral during evaluation for congestive heart failure. There was no other evidence of cardiopulmonary disease. The collateral was successfully closed with multiple coils delivered sequentially. We describe the issues associated during closure of the aortopulmonary collateral in this case. To the best of our knowledge, this is the first reported case of large aortopulmonary collateral presenting with heart failure in an otherwise structurally normal heart that was closed successfully with multiple coils delivered sequentially.

## INTRODUCTION

Aortopulmonary collaterals are a form of alternative blood supply in congenital heart disease with decreased pulmonary blood flow, e.g., tetralogy of Fallot (TOF), ventricular septal defect (VSD) with pulmonary atresia, etc.[1] Aortopulmonary collaterals are also noted in neonates, especially premature ones with bronchopulmonary disorder.[2-4] These collaterals are usually small and rarely cause symptoms. They usually regress on their own after treatment of the primary disorder and rarely need intervention.[5-7] We describe a case of large aortopulmonary collateral presenting as congestive heart failure in an otherwise structurally normal heart that was closed successfully using multiple coils delivered sequentially.

## CASE REPORT

A 7-month-old girl, weighing 5.5 kg, was found to have poor weight gain and shortness of breath from the second month of life. She was delivered at term, without any perinatal complications with a birth weight of 2.7 kg. At presentation her clinical examination showed mild cardiomegaly and left ventricular third heart sound. The precordial examination was otherwise normal. She had a faint continuous murmur on the left infrascapular area. Chest X-ray showed mild cardiomegaly with normal pulmonary blood flow, and no evidence of pulmonary dysplasia. Echocardiogram showed left atrial (LA) and left ventricular (LV) dilatation and a bicuspid aortic valve without any aortic stenosis or aortic regurgitation. The interatrial septum and interventricular septum were intact and there was no patent ductus arteriosus. Abdominal aortic doppler showed pan-diastolic flow reversal. A 4.6-mm abnormal vessel was seen arising from the descending aorta coursing towards left lung. The pulmonary and systemic venous drainages were normal.

**Figure 1:**
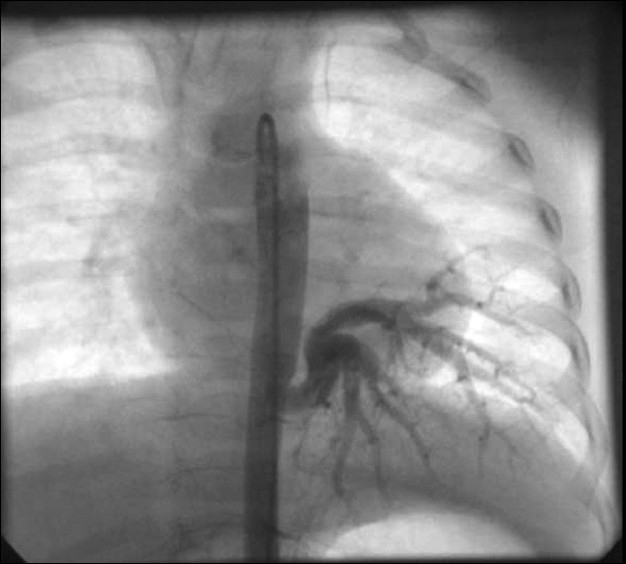
Aortogram in AP view showed a large aortopulmonary collateral supplying the left lower lobe

**Figure 2:**
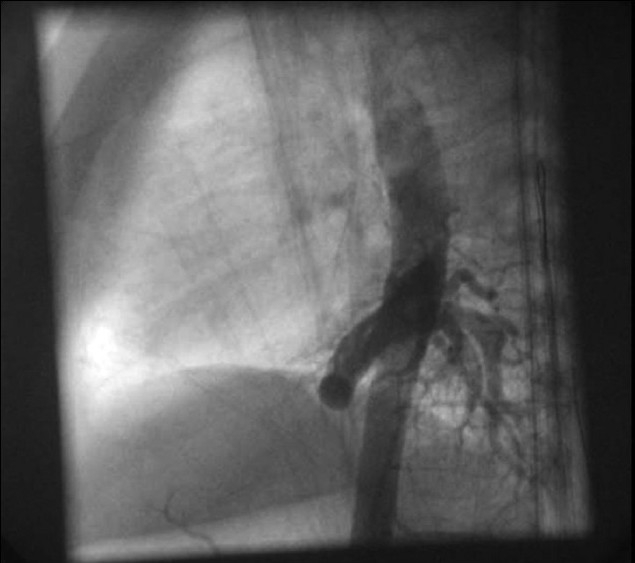
Aortogram in lateral view showed a large aortopulmonary collateral supplying the left lower lobe

**Figure 3:**
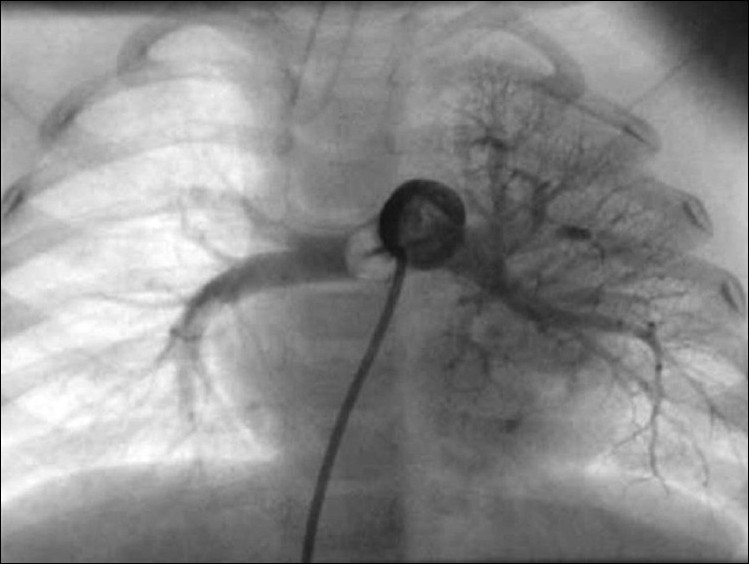
Pulmonary angiogram in AP view showing normal branching pattern; the left lower lobe is also being supplied by the pulmonary artery

**Figure 4:**
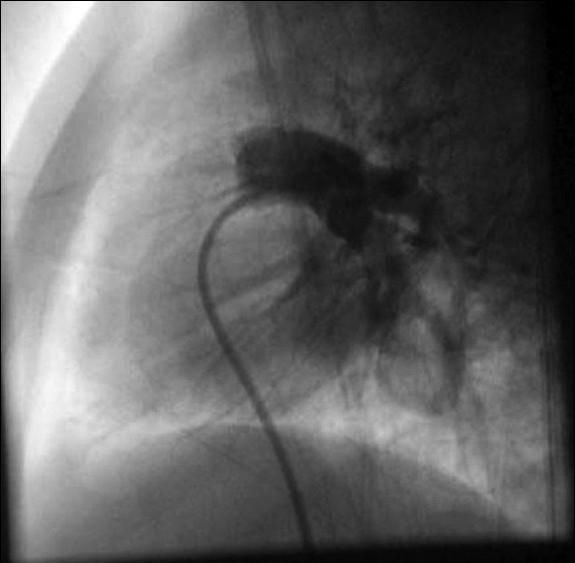
Pulmonary angiogram in AP and lateral views showing normal branching pattern; the left lower lobe is also being supplied by the pulmonary artery

At cardiac catheterization, the pulse pressure was wide (systemic arterial pressure of 110/50 mmHg with pulse pressure of 60 mmHg). The pulmonary artery pressure was normal (20/12 mm Hg; mean-15 mmHg). Descending aortic angiogram and subsequent selective angiogram in anterio-posterior (AP) and lateral views showed a large 5.8-mm aortopulmonary collateral arising from T 7-8 level supplying the whole left lower lobe [Figures 1 and 2]. The levo-phase of the angiogram showed the pulmonary venous return to the left atrium. Pulmonary artery angiogram, in AP and lateral view [Figures 3 and 4], revealed dual blood supply to the left lower lobe, and levo-phase showed the pulmonary venous drainage to the LA, thus ruling out lobar sequestration.

**Figure 5:**
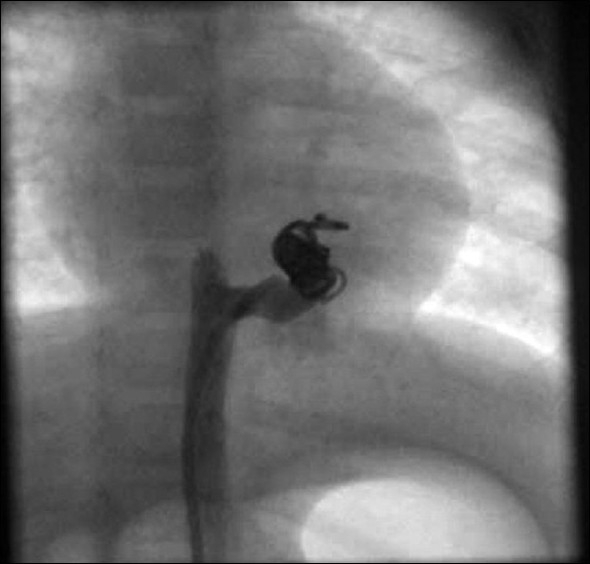
Selective angiogram of the aortopulmonary collateral after the procedure, showing complete closure

The collateral was closed with six, 0.038” 5 cm × 5 mm coils (Cook Inc., Bloomington, IN) delivered through a 5F Multipurpose A catheter (Cook Inc.) sequentially. After the sixth coil deployment, angiogram showed complete occlusion of the collateral [Figure 5].

The post-procedure course was uneventful. The patient was discharged on the second day after the procedure. Pre-discharge echo showed normalization of LA and LV sizes.

## DISCUSSION

In a study on rats, Ruiter and colleagues demonstrated that aortopulmonary collaterals are persistent ventral segmental arteries that connect the pulmonary plexus with the dorsal aortae early in fetal life.[8] However, what is the stimulus for their persistence in the postnatal life is not known.

Collateral arteries have also been described as persistent, abnormally dilated arteries that connect the bronchial arteries to the pulmonary circulation secondary to external factors such as hypoxia, trauma or inflammation. Hypoxia, in cases of decreased pulmonary blood flow; and hypoxia, barotrauma and inflammation in cases of bronchopulmonary dysplasia and other cases of neonatal pulmonary injury, act as the stimuli for the persistence of the collaterals in the postnatal life.[4,6]

In our case, the child was born at term, with birth weight of 2.7 kg and had uncomplicated neonatal period. Features suggestive of congestive heart failure started at about the second month of life, similar to post-tricuspid left-to-right shunts. She did not have any features suggestive of bronchopulmonary dysplasia. Again the child did not have any cardiac lesion causing decreased pulmonary blood flow. Hence the etiology of development and persistence of large isolated aortopulmonary collateral in our case remain obscure.

The clinical course of our case was similar to that of a large post-tricuspid left-to-right shunt, presenting as congestive heart failure after decrease in the neonatal pulmonary hypertension. Since the aortopulmonary collateral was symptomatic, it needed closure. The options available were, either simultaneous delivery of multiple coils or use of vascular plug.[9,10] For simultaneous delivery of multiple coils, a 7F sheath was needed. However, in a small child with a weight of 5.5 kg, it would have been difficult without causing damage to femoral artery. The other option was to use the second-generation vascular plug (Amplatzer vascular plug II), which could have been used with a 5F sheath. However, because of the cost factor, this was not considered. Hence we chose sequential delivery of 0.038” 5 cm × 5 mm coils through a 5F Multipurpose A catheter. A total of 6 such coils were needed to close the collateral completely [Figure 5]. After the procedure, there was no loss of femoral pulse.

## CONCLUSION

Even though cases of closure of aortopulmonary collaterals with coils have been reported, all except one reported by Ralf Holzer *et al*. were associated with bronchopulmonary dysplasia. Again, none of the cases described presented as congestive heart failure. To the best of our knowledge, this is the first case of multiple coils closure deployed sequentially in a patient with isolated aortopulmonary collateral presenting as congestive heart failure.
